# Self-Powered Implantable Skin-Like Glucometer for Real-Time Detection of Blood Glucose Level In Vivo

**DOI:** 10.1007/s40820-017-0185-x

**Published:** 2018-01-04

**Authors:** Wanglinhan Zhang, Linlin Zhang, Huiling Gao, Wenyan Yang, Shuai Wang, Lili Xing, Xinyu Xue

**Affiliations:** 1 College of Sciences, Northeastern University, Shenyang, 110004 People’s Republic of China; 2 College of Life and Health Sciences, Northeastern University, Shenyang, 110004 People’s Republic of China

**Keywords:** Diabetes, Biosensor, Electronic-skin, Self-powered, Glucose detection, Implantable electronics

## Abstract

Implantable bioelectronics for analyzing physiological biomarkers has recently been recognized as a promising technique in medical treatment or diagnostics. In this study, we developed a self-powered implantable skin-like glucometer for real-time detection of blood glucose level in vivo. Based on the piezo-enzymatic-reaction coupling effect of GOx@ZnO nanowire, the device under an applied deformation can actively output piezoelectric signal containing the glucose-detecting information. No external electricity power source or battery is needed for this device, and the outputting piezoelectric voltage acts as both the biosensing signal and electricity power. A practical application of the skin-like glucometer implanted in mouse body for detecting blood glucose level has been simply demonstrated. These results provide a new technique path for diabetes prophylaxis and treatment.

**Electronic supplementary material:**

The online version of this article (10.1007/s40820-017-0185-x) contains supplementary material, which is available to authorized users.

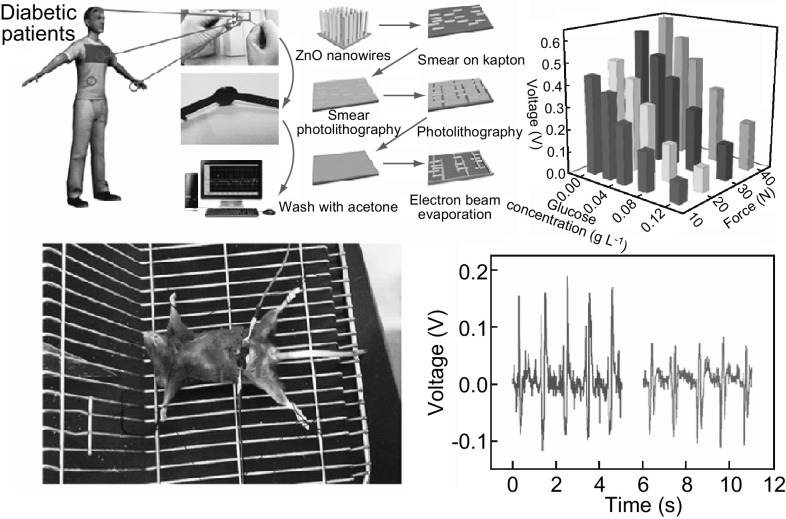

## Highlights

- Self-powered implantable skin-like glucometer for real-time detection of blood glucose level in vivo was fabricated.
- The distinct interdigitated electrode ensures piezo-potential of individual nanowires in the same direction. Piezo-biosensing process does not require an external power supply. And the piezoelectric-enzyme-reaction coupling effect is proposed.
- The device can run well in live mouse and detect in real time the glucose level.

## Introduction

Diabetes mellitus (DMs), a group of metabolic diseases, in which there are high blood glucose levels over a prolonged period, is among the series of problems challenging the human health. If left untreated, diabetes can cause a series of complications, such as blindness, diabetic foot, renal failure, heart disease, and stroke. Since diabetes may exist without clinical manifestations at the early stage, symptomatic cases may be difficult to detect. Continuous blood glucose monitoring in daily life is necessary for prophylaxis and early treatment of diabetes [1, 2]. At present, the most commonly utilized approach in medical field is blood test. Although the test of blood sample in vitro can directly monitor blood glucose with great precision, some shortcomings still exist. One is the difficulty of realizing real-time monitoring, and the other is that a blood test needs too many steps, including blood sampling and chemical analysis [3].

With the rapid development of semiconductor industry and information technology, wearable electronics (e.g., electronic skin) with various functions have attracted a wide range of attention. Recently, the wearable skin-like biological sensors have emerged as effective noninvasive transducers of human physiological signals in healthcare [4–11]. However, there are still lots of challenges of skin-like biosensors for implanting in human body. First of all, as the skin-like biosensor is implanted inside human body, the material must be flexible and soft, and the biosensing functions must have clinical significance [12, 13]. On the other hand, energy consumption is a bottleneck problem for the skin-like biosensors to be used inside the body in large scale and long term. Although common electricity power sources (e.g., batteries) have high energy density, they are not suitable for integrating in the skin-like biosensing systems. The rigid shell of the battery and leakage risk are opposite to the implanting requirements, and the charging and replacing processes of the battery are inconvenient in the inner body environment. Our previous work shows that the piezo-enzymatic-reaction coupling effect of GOx@ZnO (GOx: glucose oxidase) nanowires can realize detecting glucose concentration without battery, but the device structure and implanting operation need to be developed [14].

**Fig. 1:**
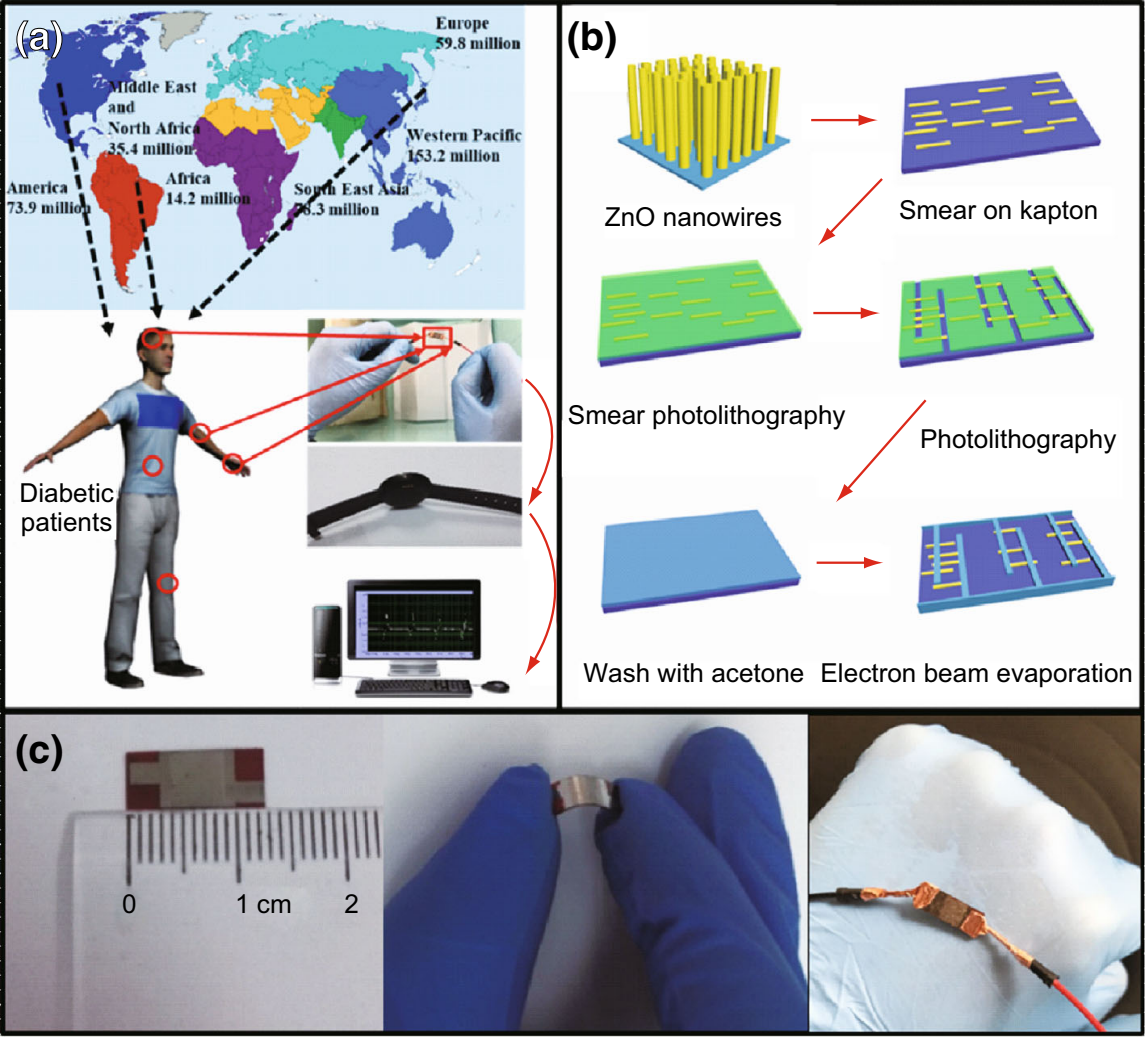
Potential application, device architecture, material system, and experimental design of self-powered implantable skin-like glucometer. **a** Potential application. **b** Device architecture, material system, and fabrication procedure. **c** Optical images of the device

The World Health Organization (WHO) predicts that there will be 300 million people having diabetes by the year 2025 (Fig. 1a). The continuous monitoring of blood glucose concentration is strongly requested [15–18]. Here, a self-powered implantable skin-like glucometer for real-time detection of blood glucose level has been realized based on the piezo-enzymatic-reaction coupling process of horizontally aligned GOx@ZnO nanowire arrays [19–22]. The device can actively output piezoelectric voltage under applied deformation, and the outputting piezoelectric voltage contains the information of glucose concentration inside the body [23–26]. The outputting piezoelectric voltage can act as not only the electricity power for driving the device but also the biosensing signal. In this process, no external electricity power source or battery is needed [27–29]. A practical application of the device implanted in mouse body for detecting its blood glucose level without any external electricity power has been simply demonstrated. Our results could provoke a possible new research field of self-powered/real-time diabetes monitoring and diagnosis.

## Experimental

### Fabrication of the Self-Powered Implantable Skin-Like Glucometer

All the analytical-grade chemical reagents for synthesizing GOx@ZnO nanowire arrays were supplied by Sinopharm Chemical Reagent Co. Ltd.

A seed-assisted hydrothermal method was used to align ZnO nanowire arrays vertically on the Ti substrate [30–32]. Firstly, the Ti substrate (thickness of 100 µm) was washed for several times with alcohol and DI water to remove surface impurities, and dried in an oven at 60 °C. Zn(CH_3_COO_2_)_2_·H_2_O solution (10 mM in ethanol) was dropped onto the pre-cleaned Ti substrate and blown dry with nitrogen gas. This process was repeated for several times. Secondly, in order to produce the seed layer, the Ti substrate was conducted with an annealing treatment at 350 °C for 20 min. Thirdly, 0.416 g of Zn(NO_3_)_2_·6H_2_O was dissolved in 38 mL of DI water and stirred for 10 min at room temperature. Two milliliters of NH_3_·H_2_O was added into the solution under continuous stirring, and the Ti substrate was immersed in the solution. After that, the reaction beaker was sealed and maintained at 90 °C for 24 h. Finally, Ti substrate was taken out, rinsed with ethanol/DI water, and dried at 60 °C.

As the flexible substrate for supporting the device, a Kapton board was cleaned with alcohol and DI water for several times and dried at 60 °C. The horizontally aligned ZnO nanowire arrays (overwhelmed down on Ti substrate) were carefully transferred to the Kapton substrate, while ensuring that all the nanowires were in the uniform direction. The Kapton substrate with ZnO nanowires was patterned by photolithography using positive photoresist. The 200 nm-thick layer of Ti as electrode was subsequently deposited by electron beam evaporation at room temperature. In order to remove the abundant Ti and photoresist, the Kapton substrate was immersed in an organic solvent, rinsed with deionized water, and dried at 60 °C. Finally, ZnO nanowires were modified with GOx (supplied by Sigma Chemical Co. St. Louis, MO, USA). 0.5 mL of the configured GOx aqueous solution (10 g L^−1^) was slowly dropped onto the surface of ZnO nanowires. The device was placed in a dry and ventilated place for 2–3 h [33]. This process was repeated four times to complete the attachment of GOx [34]. Finally, each device should be covered with 20 mg GOx on 0.4 cm^2^ area. In the previous work, ZnO has proven to be nontoxic and can work well in the human body environment, as well as the substrate of Kapton and the electrode material of Ti [35–39]. Thus, the whole material system in our device is nontoxic and biocompatible.

### Characterization and Measurement

The crystal phase of ZnO nanowire arrays was characterized by X-ray diffraction (XRD, D/max 2550 V, Cu Kα radiation). The morphology and microstructure of the nanowires and the skin-like glucometer device were investigated by a scanning electron microscope (SEM, JEOL JSM-6700F) and transmission electron microscope (TEM, JEOL JEM-2010).

The skin-like glucometer was completely submerged in the aqueous solution of glucose. A programmed motor which could provide a constant force for simulating body motion was used to drive the device with a fixed frequency. The force was applied on the center of the device (the supporters were on the sides), and the device can be bent. A force sensor was used to measure the magnitude of the force. The outputting piezoelectric voltage was measured using a low-noise preamplifier (Model SR560, Stanford Research Systems).

### Experiment In Vivo

The skin-like glucometer was implanted into the mouse body, and the surgery process was as follows. The mouse was injected with anesthetic for the following surgery. The device was implanted into the mouse’s abdomen under the skin, where the biosensor unit can directly contact with the mouse blood. The edges of the device were connected with two electrode poles to receive the outputting piezoelectric voltage generated by the device [40]. After that, we slowly injected 5 mL of glucose solution (0.045 g L^−1^) into the mouse abdomen using a medical syringe within about 5 s. All of the procedures in this study conformed to the regulations for the administration of affairs concerning experimental animals and were approved by Northeastern University.

## Results and Discussion

Figure 1a shows the experimental design of this work. Figure 1b schematically illustrates the material system and fabrication procedure of self-powered implantable skin-like glucometer [40, 41]. The device is assembled by positioning parallel ZnO nanowires (with uniform direction) onto the Ti interdigitated electrodes on a soft Kapton substrate. To ensure the outputting piezoelectric voltage, the distance between neighboring electrode couples is 20 μm, larger than the length of ZnO nanowire (~ 12 μm) [42]. Figure 1c shows the optical images of the device. The thin device is 0.4 × 1.3 cm^2^ in size and can be easily bended. The thickness of the Kapton film is 250 μm, and the overall thickness of the device is less than 260 μm. Compared with our previous work [14], the device structure and application are greatly improved in this study. The new substrate is more flexible and stretchable. The nanowires are horizontally aligned on the substrate instead of vertical arrangement, which can facilitate the biosensing process. The new device is very small and suitable for embedding into the organism and can be implanted into mouse body to detect glucose concentration in vivo.

**Fig. 2:**
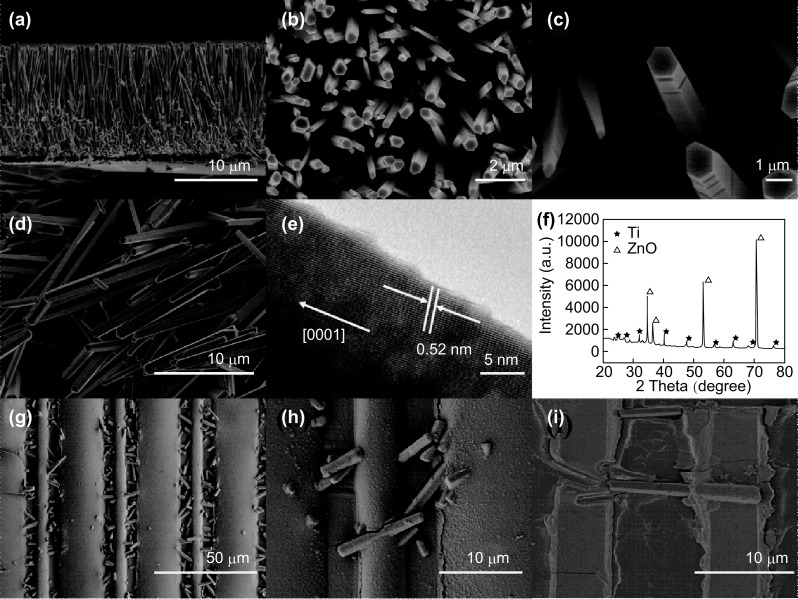
**a** SEM image of as-grown ZnO nanowire arrays on Ti substrate on the side view. **b**, **c** Top-view SEM images of as-grown ZnO nanowire arrays. **d** SEM image of ZnO horizontally aligned nanowire arrays (overwhelmed in one direction) before smearing on Kapton substrate. **e** High-resolution TEM image of one single ZnO nanowire. **f** XRD pattern of ZnO nanowires. **g** Top-view SEM image of the device (before washing with acetone). **h**, **i** Enlarged view of the device

The morphology and structure of ZnO nanowire arrays and self-powered implantable skin-like glucometer are shown in Fig. 2. Figure 2a shows the SEM image of as-grown ZnO nanowire arrays on Ti substrate on the side view, showing that ZnO nanowires are vertically aligned on the substrate. The average length of the nanowires is about 12 μm. Figure 2b, c shows the top-view SEM images of as-grown ZnO nanowire arrays, showing that ZnO nanowires have a uniform distribution on the substrate with the same growth direction and the average diameter of the nanowires is about 160 nm. Figure 2d shows the SEM image of horizontally aligned ZnO nanowire arrays (overwhelmed in one direction) before smearing on Kapton substrate [43]. The same growth direction of the overwhelmed nanowire arrays ensures that the crystallographic orientations of the nanowires in the following device are aligned along the same direction. Consequently, the polarities of the induced piezo-potential of individual nanowires are also in the same direction, leading to a macroscopic potential contributed constructively by all of ZnO nanowires. Figure 2e shows the high-resolution TEM image of one single ZnO nanowire. It can be observed that the lattice spacing is 0.52 nm, consistent with (001) crystal plane of wurtzite structural ZnO [44, 45]. The XRD pattern of ZnO nanowires is shown in Fig. 2f, and the sharp diffraction peaks indicate good crystalline quality. All the diffraction peaks can be indexed to Ti (JCPDS card no. 44-1294) and the hexagonal wurtzite structure of ZnO (JCPDS card no. 36-1451). The top-view SEM image of the device (before washing with acetone) and the enlarged view are shown in Fig. 2g, h, respectively. ZnO nanowires locate across Ti interdigitated electrodes. Figure 2i shows that after removing the photoresist with acetone one single ZnO nanowire bridges the two electrodes in one electrode couple.

**Fig. 3:**
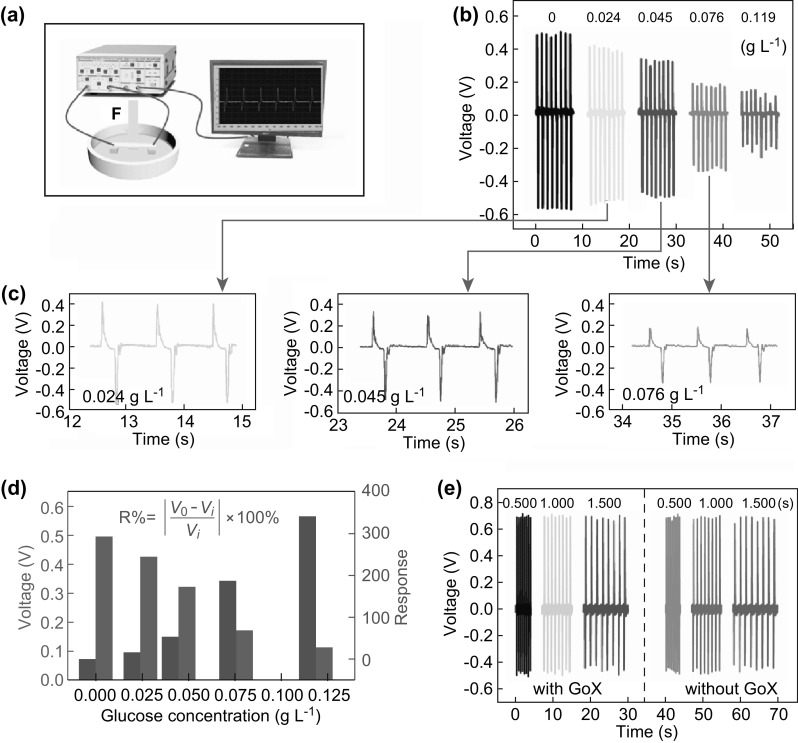
**a** The measurement system. **b** The outputting piezoelectric voltage of self-powered implantable skin-like glucometer in different concentrations of glucose solution. The applied force is 21 N. **c** The enlarged views of the outputting piezoelectric voltage against 0.024, 0.45, and 0.076 g L^−1^ glucose solution. **d** The outputting piezoelectric voltage and the response of the device against different glucose solution concentration. **e** The piezoelectric voltage of the skin-like glucometer with and without GOx modification in air with different force frequencies

Figure 3 shows the piezoelectric-biosensing performance of self-powered implantable skin-like glucometer. The device is completely immersed in the aqueous solution of glucose, as shown in Fig. 3a [46]. Under an applied deformation, the device (being bended) can transfer the mechanical energy to piezoelectric voltage that carries the information of glucose concentration. No other electric power is needed during this process, and the outputting piezoelectric voltages can act as both the power source and the biosensing signal. Figure 3b shows that the outputting piezoelectric voltage of the device decreases with increasing glucose concentration. As the concentrations of glucose are 0, 0.024, 0.045, 0.076, and 0.119 g L^−1^, the voltage of the device is about 0.49, 0.42, 0.32, 0.17, and 0.11 V, respectively. The enlarged views of the outputting piezoelectric voltage against 0.024, 0.045, and 0.076 g L^−1^ are shown in Fig. 3c. These results indicate that the device can monitor the glucose concentration [47, 48].

The response of the device can be calculated from Eq. 1 [49]:

1 $$R\% = \left| {\frac{{V_{0} - V_{i} }}{{V_{i} }}} \right| \times 100\%$$

where *V*_0_ and *V*_i_ are the outputting piezoelectric voltage of the device in pure water and glucose aqueous solution, respectively. Figure 3d shows that the response of the device against glucose concentrations of 0.024, 0.045, 0.076, and 0.119 g L^−1^ is 16, 53, 188, and 340, respectively. Obviously with the increasing concentration of glucose solution, the outputting piezoelectric voltage is reduced. On the contrary, the response increases with increasing glucose concentration. This device can sensitively detect glucose concentration, and no external electricity power is needed in the piezo-biosensing process. The outputting piezoelectric voltage is linearly dependent on glucose concentration (0.024–0.119 g L^−1^), as shown in Fig. S4. The limit of detection (LOD; the signal-to-noise ratio is 3:1) can be calculated to be about 0.019 g L^−1^ [50]. Figure 3e exhibits the piezoelectric voltage of ZnO nanowires with and without GOx modification in air with different force frequencies. It can be seen that the outputting piezoelectric voltage arises from the piezoelectric effect of ZnO nanowires.

**Fig. 4:**
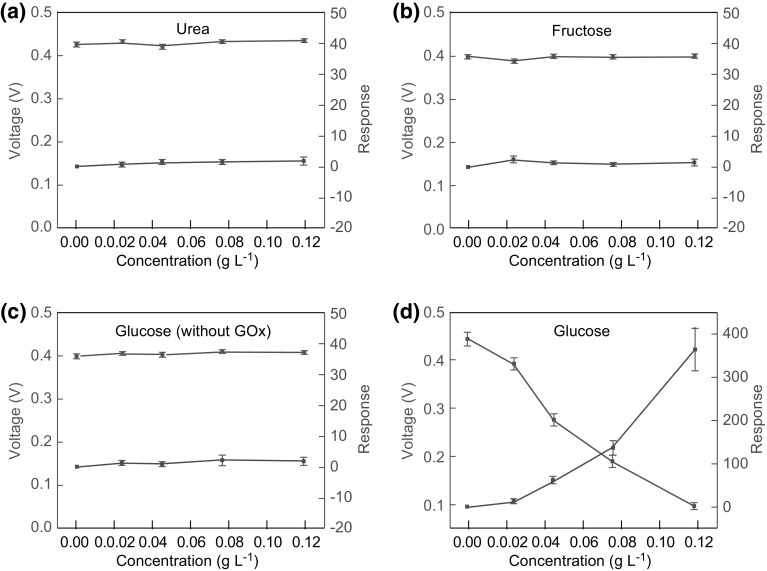
**a**, **b** The outputting piezoelectric voltage and response of the self-powered implantable skin-like glucometer against urea and fructose. **c** The outputting piezoelectric voltage and response of the device without GOx modification against glucose. **d** The outputting piezoelectric voltage and response of the device with GOx modification against glucose

When the self-powered implantable skin-like glucometer is practically used inside a human body, the various kinds of electrolytes and metabolites in body fluid may influence the biosensing accuracy [51]. Therefore, selectivity is an important parameter of the device to detect specific target analytes. Figure 4 shows the selectivity test of the device against three kinds of common ingredients in the body fluid (glucose, fructose, and urea) [52, 53]. The response of the device against fructose and urea is very small (Fig. 4a, b). Figure 4c, d shows that the change in outputting piezoelectric voltage is due to the reactions between glucose and oxidase, rather than the influence of water. The results suggest that the device has a good selectivity for detecting glucose. The influence caused by other electrolytes and metabolites can probably be neglected.

Figure S1 shows the sensing performance of self-powered implantable skin-like glucometer under different forces (14, 22, 31, and 40 N). The applied forces have the same frequency of 1.0 Hz. Under the force of 14 N, the outputting piezoelectric voltage of the device against 0, 0.024, 0.045, 0.076, and 0.119 g L^−1^ glucose solutions is 0.45, 0.39, 0.27, 0.18, and 0.09 V, respectively (Fig. S1a). Under the force of 22 N, the outputting piezoelectric voltage is 0.49, 0.42, 0.32, 0.17, and 0.11 V, respectively (Fig. S1b). Under the force of 31 N, the outputting piezoelectric voltage is 0.60, 0.50, 0.41, 0.29, and 0.16 V, respectively (Fig. S1c). Under the force of 40 N, the outputting piezoelectric voltage is 0.64, 0.56, 0.46, 0.34, and 0.22 V, respectively (Fig. S1d). As the applied force increases, the outputting piezoelectric voltage increases (Fig. S1e). Interestingly, as shown in Figure S1f, the response of the device under different applied forces is very close. This feature can facilitate the practical applications of our device.

For showing the flexibility of self-powered implantable skin-like glucometer, the biosensing performance at different bending angles is presented in Fig. S2 [54]. The bending angle is controlled by the moving distance of the stepper motor. Figure S2a shows the outputting piezoelectric voltage of the device against 0.045 g L^−1^ glucose solution at different bending angles. The relationship between outputting piezoelectric voltage and bending angle is shown in Fig. S2b. When the bending angle of the skin-like glucometer is 0°, 10°, 20°, and 30^∘^, the outputting piezoelectric voltage in 0.045 g L^−1^ glucose solution is 0.27, 0.32, 0.41, and 0.45 V, and the voltage in 0 g L^−1^ glucose solution is 0.45, 0.49, 0.58, and 0.63 V, respectively. As the bending angle increases, the response decreases (Fig. S2c). It may be attributed to the damage on GOx@ZnO nanowires as the bending angle increases.

The stability of self-powered implantable skin-like glucometer is shown in Fig. S2d. The glucose concentration is 0.045 g L^−1^; the applied force is 21 N; and the bending angle is kept at 20^∘^. After bending for 100, 200, 300, 400, 500, 600, 700, 800, and 900 times, the response is 47, 51, 52, 49, 49, 48, 48, 53, and 51, respectively. These results indicate that the device has high stability.

**Fig. 5:**
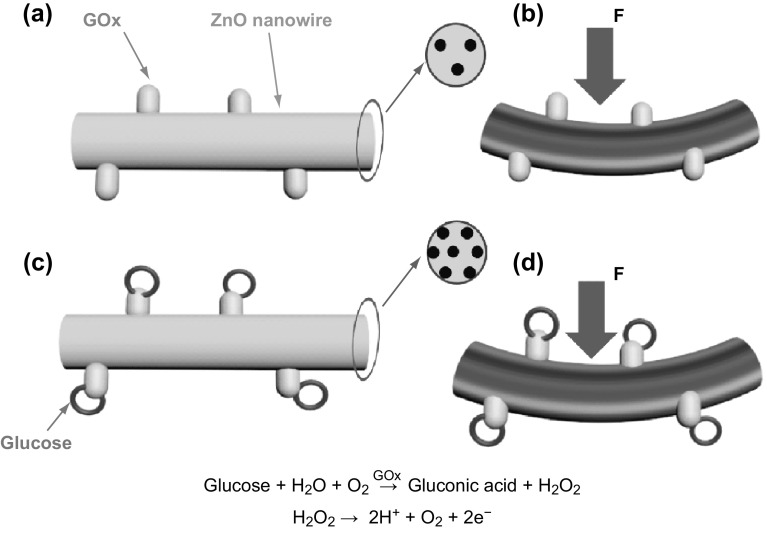
Working mechanism of self-powered implantable skin-like glucometer. **a** GOx/ZnO nanowires in pure water without applied deformation. **b** The piezoelectric output of GOx/ZnO nanowires in pure water under applied deformation. **c** GOx/ZnO nanowires in glucose aqueous solution without applied deformation. **d** The piezoelectric output of GOx/ZnO nanowires in glucose aqueous solution under applied deformation

The working principle of self-powered implantable skin-like glucometer is schematically illustrated in Fig. 5. The working mechanism is based on the coupling between enzymatic reaction and piezo-screening effect of ZnO nanowires. In pure water, no reactions take place on the surface of GOx@ZnO nanowire, and the surface carrier density of the nanowire is low, as shown in Fig. 5a. Under applied deformation, GOx@ZnO nanowire can create piezoelectric potential with weak piezo-screening effect, and the outputting piezoelectric voltage is high (Fig. 5b). When the device is immersed in the glucose solution, the GOx attached on ZnO nanowire surface reacts with glucose, as shown in Fig. 5c. On the first step, gluconic acid and H_2_O_2_ are generated as the following reaction [55–57]:

2 $${\text{Glucose}} + {\text{H}}_{2} {\text{O}} + {\text{O}}_{2} \mathop{\longrightarrow}\limits^{\text{GOx}}{\text{Gluconic}}\;{\text{acid}} + {\text{H}}_{2} {\text{O}}_{2}$$

It has been reported that H_2_O_2_ can transfer electrons to ZnO nanowire and produce hydrogen ions as the following decomposition reaction [58]:

3 $${\text{H}}_{2} {\text{O}}_{2} \to 2{\text{H}}^{ + } + {\text{O}}_{2} + 2{\text{e}}^{ - }$$

In this process, electrons are absorbed on the surface of the nanowire, which can increase the surface carrier density [59]. And H ^+^ ions can also be absorbed on the surface of the nanowires, acting as extra carriers [14, 55]. Under applied deformation, the outputting piezoelectric voltage of the nanowire decreases due to the strong piezo-screening effect from the large amount of H^+^ and e^−^ on the surface of the nanowire (Fig. 5d).

To further confirm the mechanism, the response of the device against H_2_O_2_ and CH_3_COOH (H^+^) solutions has been tested as shown in Fig. S3. As the concentration of H_2_O_2_ is 0, 0.024, 0.045, 0.076, and 0.119 g L^−1^, the outputting piezoelectric voltage of the device is about 0.46, 0.29, 0.19, 0.13, and 0.07 V (Fig. S3a), and the response is about 0, 55, 133, 258, and 505 (Fig. S3c), respectively. As the concentration of CH_3_COOH is 0, 0.024, 0.045, 0.076, and 0.119 g L^−1^, the outputting piezoelectric voltage of the device is about 0.601, 0.479, 0.435, 0.358, and 0.301 V (Fig. S3b), and the response is about 0, 25, 38, 67, and 99 (Fig. S3d), respectively. It can be seen that the outputting piezoelectric voltage of the device decreases with increasing concentration of H_2_O_2_ or H^+^. To easily understand all the above experimental results and compare them, Table S1 lists the information.

**Fig. 6:**
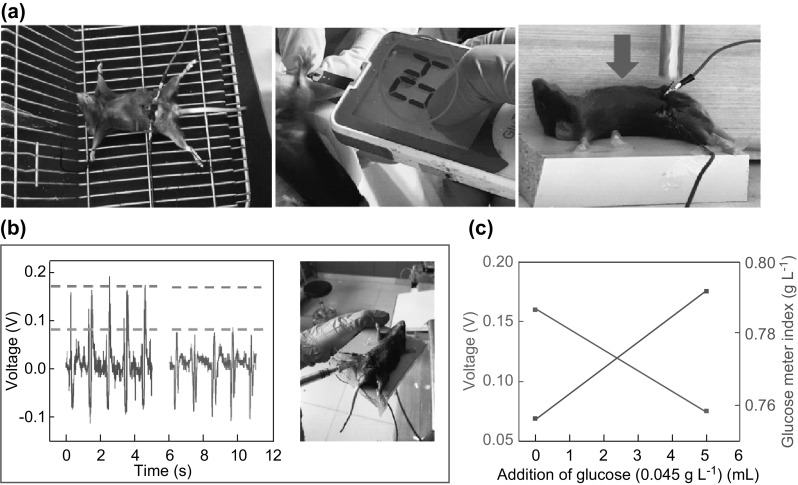
Practical application of self-powered implantable skin-like glucometer implanted in a mouse body for detecting blood glucose concentration without any external electricity power source. **a** Implanting the device into the mouse and measurement set. **b** The outputting piezoelectric voltage changes before and after injection of glucose solution. **c** The outputting piezoelectric voltage and glucose meter index of the device before and after the injection of glucose solution

Figure 6 shows the practical application of self-powered implantable skin-like glucometer implanted in a mouse body for detecting blood glucose concentration without any external electricity power source [60–62]. The device is implanted into the mouse body by surgery process. After that, a constant force of 4 N controlled by a programming motor is applied on the device in the soft mouse abdomen (Fig. 6a). As the mouse is in anesthesia and cannot have movement, the motor is used to provide force to drive the device. The stepper motor keeps providing constant force (both the frequency and magnitude kept constant) on the device throughout the whole measurement. Thus, the applied strain of the device before and after glucose injection keeps the same. The response of the device is presented in Fig. 6b, c. It was shown that our devices are still able to respond sensitively to the changes in glucose concentration in the biological environment. Without the injection of glucose solution, the blood glucose concentration of mouse is 0.756 g L^−1^ (measured by a commercial blood glucometer), and the outputting piezoelectric voltage is around 0.16 V. After injecting 0.045 g L^−1^ glucose aqueous solution (5 mL) into the mouse abdomen, the outputting piezoelectric voltage decreases to 0.075 V. The commercial glucometer shows that the blood glucose concentration of mouse is 0.792 g L^−1^. These results can simply and roughly demonstrate that our device can work inside the mouse body and detect changes in blood glucose concentration.

Our device can harvest tiny mechanical energy of body movement, such as finger pressing or arm flexing. These motions can easily provide enough force (several tens of newton) for driving the device. It should be noted that the device needs to work during the moment of movement and cannot provide its own energy during sleep or rest. In our experiment, the piezoelectric voltage of the device inside the mouse body needs to be tested by external voltmeter. And the device needs to be driven by external applied deformation. Further work can be focused on integrating the signal processor, wireless signal emitter, and mechanical unit with the device, and the whole system can work independently inside the body.

It should also be pointed that the device is still invasive since a “surgery” process is conducted to implant this device into a mouse under the skin, which is against the future demand of noninvasive glucose monitoring. Thus, there are two development direction of the device in the future. One is that the device will detect body fluid outside the skin (tear, saliva, and so on) by improving performance. The working range of our device (0.024–0.119 g L^−1^) is below the concentration found in blood (0.36–5.4 g L^−1^). By considering the size of the device, its biocompatibility and problems with long-term usage of enzymatic sensors, we could probably fabricate a contact lenses glucose sensor (concentration in tears 0.018–0.108 g L^−1^) in the future study [17]. The other one is that the overall size of the sensor system will be integrated into the micro-nano-level, and it can be implanted into the human body through a simple method without surgery. It should also be noted that the Kapton film is relatively thick, and this Kapton film cannot be conformably implanted inside the body. The material system and the device structure have not reached the level for practical application. In the future work, we will print the device on much conformable substrates, such as PDMS.

## Conclusion

In summary, we developed a flexible self-powered implantable skin-like glucometer for real-time monitoring blood glucose level in body for the prophylaxis and treatment of diabetes. The working mechanism is based on the piezo-enzymatic-reaction coupling effect of GOx@ZnO nanowire arrays. Under applied force, the device can convert the mechanical energy into piezoelectric impulse, which is significantly influenced by blood glucose concentration. The outputting piezoelectric voltage acts as both the biosensing signal and electricity power. A practical application of the device implanted inside mouse body to real-time monitor the blood glucose concentration has been demonstrated. Our work can probably provide a new path for diabetes diagnosis.

## Electronic supplementary material

Below is the link to the electronic supplementary material.

Supplementary material 1 (PDF 521 kb)
