# Supplementary material for: Self-Powered Implantable Skin-Like Glucometer for Real-Time Detection of Blood Glucose Level In Vivo
Source: Nanomicro Lett. 2018 Jan 4;10(2):32. doi: 10.1007/s40820-017-0185-x (PMC6199078; doi:10.1007/s40820-017-0185-x)
Supplement: Supplementary file 1 — Supplementary material 1 (PDF 521 kb) [file 40820_2017_185_MOESM1_ESM.pdf]

Supporting Information for

Self-Powered Implantable Skin-Like Glucometer for Real-Time Detection of Blood Glucose Level *in vivo*

Wanglinhan Zhang[1, #], Linlin Zhang[1, #], Huiling Gao[2], Wenyan Yang[1], Shuai Wang[1, *], Lili Xing[1], Xinyu Xue[1, *]

[1]College of Sciences, Northeastern University, Shenyang 110004, People's Republic of China

[2]College of life and health sciences, Northeastern University, Shenyang 110004, People's Republic of China

[#]These authors contributed equally to this work

*Corresponding authors.

E-mail: xuexinyu@mail.neu.edu.cn, s_wang@mail.neu.edu.cn

Figures and Table

Table S1 Comparison of the voltage of the device in different solutions

V \ g L^{-1}	0	0.024	0.045	0.076	0.119
Urea	0.428	0.431	0.423	0.435	0.436
Fructose	0.398	0.389	0.400	0.398	0.398
H^+	0.600	0.479	0.435	0.358	0.301
H_2O_2	0.458	0.296	0.196	0.128	0.076
Glucose	0.446	0.393	0.276	0.188	0.096

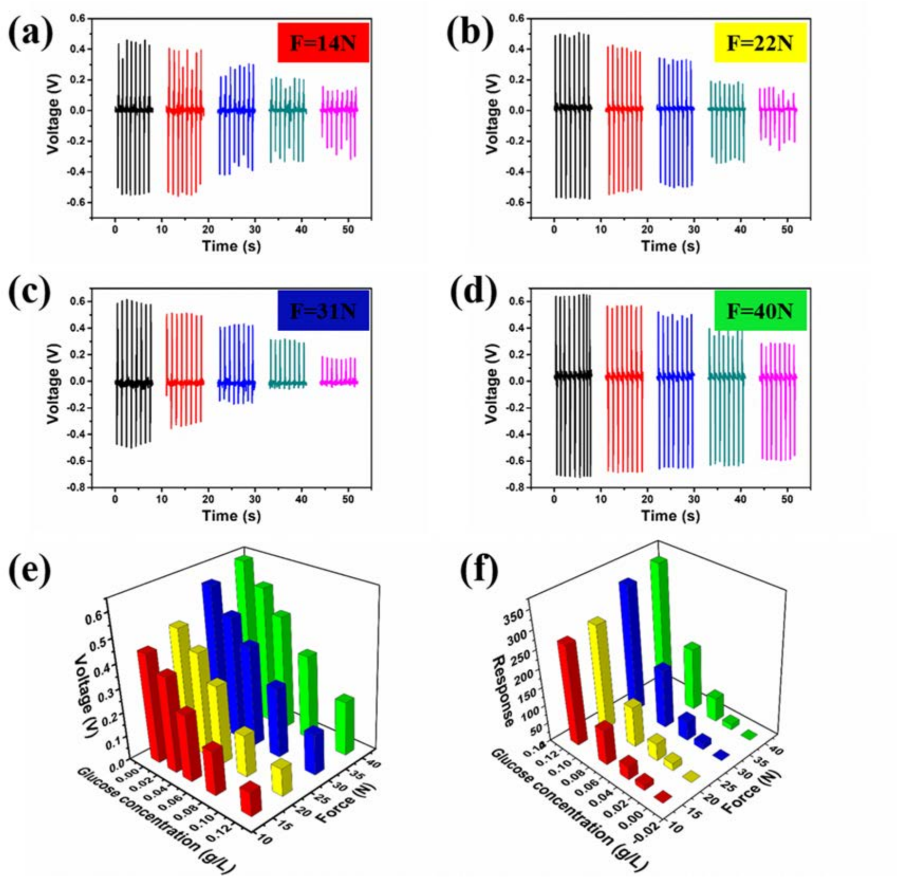

Fig. S1 a-d The outputting piezoelectric voltage of self-powered implantable skin-like glucometer with different applied forces. **e** The relationship between the outputting piezoelectric voltage and applied force. **f** The glucose response under different applied forces

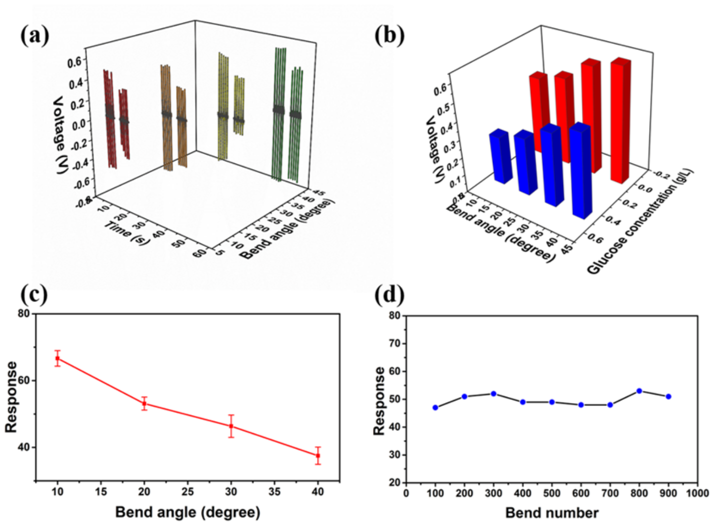

Fig. S2 Glucose response of self-powered implantable skin-like glucometer under different bending angles. **a** The outputting piezoelectric voltage of the device under different bending angles. **b** The relationship between piezo-biosensing performance and bending angle. **c** The response varies with the bending angle. **d** The relationship between response and bending number

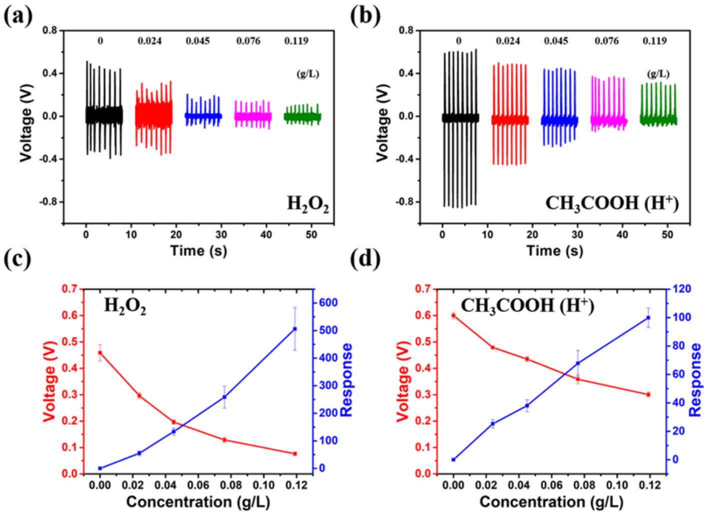

Fig. S3 **a** The outputting piezoelectric voltage of the self-powered implantable skin-like glucometer at different H_2O_2 concentration. **b** The outputting piezoelectric voltage of the device at different CH_3COOH concentration. **c** The outputting piezoelectric voltage and the response of the self-powered implantable skin-like glucometer against different H_2O_2 concentration. **d** The outputting piezoelectric voltage and the response of the device against different H_2O_2 concentration

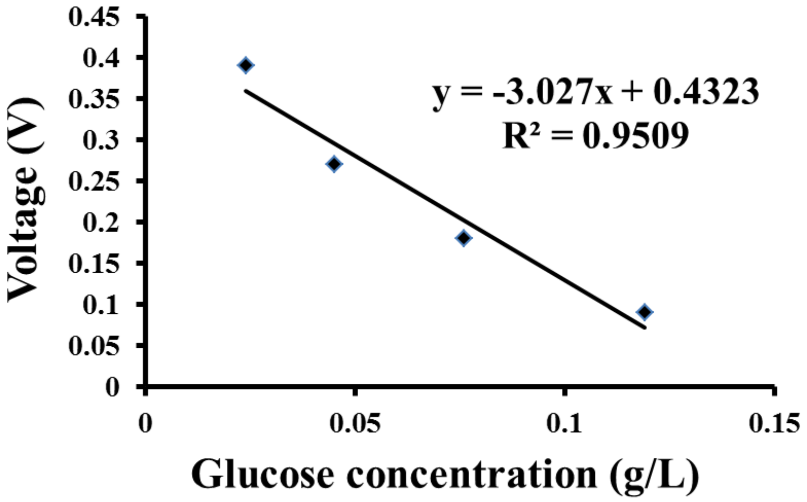

Fig. S4 Linear fitting dependencies between voltage and glucose concentration
